# Prevalence of psychological stress and adopted coping strategies among healthcare workers in King Saud Medical City (KSMC)

**DOI:** 10.1192/bjo.2021.638

**Published:** 2021-06-18

**Authors:** Mohammed Binnwejim, Atheer Alhumade

**Affiliations:** Imam Muhammed Bin Saud Islamic University

## Abstract

**Aims:**

The present study aims at investigating the level of stress among Saudi healthcare workers during COVID-19 pandemic. In addition, the present study sought to identify the coping strategies adopted by Saudi healthcare workers to alleviate the stress related to COVID-19 pandemic.

**Method:**

A descriptive cross-sectional study was performed in the period between September and November/2020. A sample of 381 healthcare workers (Physicians, nurses, and technicians) were recruited from King Saud Medical City (KSMC). Both Perceived Stress Scale-4 (PSS-4) and BRIEF-COPE scale were used to assess the levels of stress and the stress coping strategies, respectively. Descriptive statistics were used to analyze the healthcare workers’ responses about the COVID-19 related stress and their adopted coping strategies

**Result:**

The results of the study showed that there was a moderate to high level of COVID-19 related stress (11.64 ± 0.73) among the Saudi healthcare workers. In addition, it was found that planning (3.89 ± 0.61), positive reframing (3.69 ± 0.77), venting (3.39 ± 1.01), and emotional support (3.27 ± 0.63) were the most adopted coping strategies by the healthcare workers to overcome and reduce the stress levels

**Conclusion:**

The study concluded that both problem-focused and emotion-focused stress coping strategies were the most commonly adopted coping strategies among Saudi healthcare workers in KSMC. The study recommends increasing the number of the healthcare workers in the KSMC, in addition to increasing the healthcare workers’ knowledge, awareness and practice of the stress coping strategies, especially in crisis events, such as COVID-19 pandemic.

